# Paraoxonase 1 serum activity in women: the effects of menopause, the C(-107)T polymorphism and food intake

**DOI:** 10.20945/2359-3997000000130

**Published:** 2019-04-15

**Authors:** Mauren Castro Ritta, Aline Marques Baldez, Isabel Oliveira de Oliveira, Driele Neske Garcia, Paola Spiering Souza, Kelvin Ruan da Silva Andrade, Sandra Costa Valle, Simone Pieniz, Carlos Castilho Barros, Michal M. Masternak, Augusto Schneider

**Affiliations:** 1 Universidade Federal de Pelotas Faculdade de Nutrição Universidade Federal de Pelotas Pelotas RS Brasil Faculdade de Nutrição, Universidade Federal de Pelotas (UFPel), Pelotas, RS, Brasil; 2 Universidade Federal de Pelotas Instituto de Biologia Universidade Federal de Pelotas Pelotas RS Brasil Instituto de Biologia, Universidade Federal de Pelotas (UFPel), Pelotas, RS, Brasil; 3 University of Central Florida College of Medicine Burnett School of Biomedical Sciences University of Central Florida Orlando FL USA College of Medicine, Burnett School of Biomedical Sciences, University of Central Florida, Orlando, FL, USA; 4 Department of Head and Neck Surgery Greater Poland Cancer Centre Poznan Poland Department of Head and Neck Surgery, Greater Poland Cancer Centre, Poznan, Poland

**Keywords:** PON1, aging, HDL, SNPs, nutrigenetics

## Abstract

**Objective:**

The aims of this study were to investigate changes in serum paraoxonase 1 (PON1) activity in women at the pre and postmenopausal stages and its association with the *PON1* C(-107)T polymorphism and food intake profile.

**Subjects and methods:**

A cross-sectional study with female patients aged between 35 and 59 years old was conducted. Women were divided into two groups: premenopausal (n = 40) and postmenopausal (n = 36). Women enrolled in the study had serum PON1, total cholesterol, HDL, LDL, glucose and HbA1c, as well as the BMI measured. Additionally, women were genotyped for the *PON1* T(-107)C polymorphism and the food intake profile was obtained through interview.

**Results:**

Glucose (p = 0.03), HbA1c (p = 0.002) and total cholesterol (p = 0.002)concentrations were higher in post than premenopausal women, however PON1 activity was not different (p > 0.05). Carriers of the C allele had higher PON1 activity (CC: 88.9 ± 6.5 U/mL and CT: 79.9 ± 4.7 U/mL) than women of the TT genotype (66.6 ± 5.9 U/mL) (p < 0.05). However, the model predicting PON1 activity was slightly better when genotype, total fat and cholesterol content in the diet were all included.

**Conclusion:**

In sum, we observed that the *PON1* C(-107)T genotype was the major regulator of PON1 activity, and menopause had no effect on PON1 activity. The lipid and glycemic profile were altered in postmenopausal women.

## INTRODUCTION

Menopause is defined as the cessation of ovulation due to the depletion of the ovarian follicular reserve, which occurs around 50 years of age, and is characterized by the absence of menstrual cycles for at least twelve months in older women (1). As a result of the ovarian inactivity, estrogen and inhibin levels are severely reduced in postmenopausal women (1). These hormonal deficiencies lead to changes in the serum lipid profile, as well as body composition and fat distribution (2-4). Therefore, menopause onset is associated with several physiological and biochemical changes, resulting in an increased susceptibility to chronic diseases such as type 2 diabetes (4) and cardiovascular diseases (CVD) (5). The occurrence of CVD in premenopausal women is lower than in men of the same age, however, it increases in postmenopausal women to levels comparable to men (6). Therefore, it is important to understand the physiological and biochemical changes associated with menopause to prevent disease incidence in this high-risk group.

Oxidative stress plays an important role in the etiology of atherosclerosis (7). Because of its antioxidant activity, high-density lipoprotein (HDL) prevents low-density lipoprotein (LDL) particles from undergoing oxidative modification, conferring an atheroprotective effect (8). This antioxidant activity of HDL is mostly conferred by paraoxonase 1 (PON1) (9), an enzyme synthesized in the liver found circulating in plasma associated with the HDL particle (10). Serum PON1 activity is influenced by genetic and environmental factors such as atherogenic diets and smoking (11). Interesting, serum PON1 activity in females is higher than in males (11). This difference is attributed to sex steroids, since estradiol enhances PON1 activity independent of liver PON1 protein synthesis (12). Therefore, it is believed that higher PON1 activity in reproductive age women contributes to the atheroprotective effects, lowering the risk of CVD. Differences in the PON1 activity were described between pre and postmenopausal women (13), although there is no consensus regarding this issue (14). In this sense, a better understanding of how serum PON1 activity is regulated during menopause and which are the risk factors associated with serum *PON1* activity can help improve prevention of chronic diseases.

There are more than 160 single nucleotide polymorphisms (SNPs) described in the *PON1* gene (11). However, one important SNP is located in the promoter region C(-107)T (rs705379), and exerts a significant effect on PON1 serum activity (15,16) contributing with 25% of serum PON1 activity variation (17). The presence of the C allele results in serum PON1 activity up to two times higher than that observed in the presence of the T allele only (15). We have shown before that women carriers of the C allele also have higher PON1 activity than women carrying the TT genotype (16). Despite this, some suggest that PON1 activity is more important than its genetic variations in preventing CVD (18) and no association between the T(-107)C SNP and CVD has been found (19). However, information on how serum PON1 activity is regulated around menopause of women from different PON1 genotypes is still lacking.

The diet plays an important role in the prevention of CVD, as dietary intake of excess saturated fatty acids and cholesterol is associated with increased risk of CVD (20). The activity of PON1 is also modified by diet, as seen in studies that associate the consumption of cholesterol and fatty acids with PON1 activity (21). Women with a diet rich in saturated fatty acids, omega 3 and poor in omega 6 had lower PON1 activity, which was more evident in carriers of the TT genotype (16). This indicates that the effects of the diet on PON1 activity are dependent on the genotype, and given the context and the assumptions presented, could be reinforced that environmental and genetic factors contribute to the risk of CVD. However, there are few scientific reports about the association between menopause, diet, genotype and PON1 activity. Thus, this knowledge will allow more adequate intervention strategies to be implemented, with a view to preventing comorbidities associated with this stage of life, given the increase in life expectancy increases the proportion of the female population at the postmenopausal stage (22). Based on this, the aims of this study were to investigate changes in serum PON1 activity in women at the pre and postmenopausal stages and its association with the *PON1* C(-107)T polymorphism and food intake profile. Our hypothesis is that menopause will affect serum PON1 activity response to different *PON1* C(-107)T genotypes and dietary intake.

## SUBJECTS AND METHODS

### Design and population

A cross-sectional study with female patients aged between 35 and 59 years old, who attended the Units of Family Health in southern Brazil (Rio Grande, RS) in a six-month interval was conducted. The study was submitted to the Ethics Committee of the *Universidade Federal de Pelotas* and approved under the number 1.708.582. The experiment was performed in accordance to the STROBE guidelines. All participants were informed of the research objective, as well as of the methodological procedures, and signed the Consent Form.

The sample consisted of 103 women that met the inclusion criteria in the period of data collection. The inclusion criteria were to have between 35 and 59 year of age, not be receiving hormonal treatment, and for the menopausal group to be at least one year in amenorrhea. Women who used medication that compromised vascular function and the hypothalamic-pituitary-gonadal axis, pregnant women, nursing mothers and women with early menopause (less than 40 years), late (over 55 years) or from surgical procedure were excluded from the study. The sample size calculation was based on the literature review and was carried out in the OpenEpi software (online version 3.01). To detect a significant (p < 0.05) effect with power of 95% for a 13% difference in PON1 activity between pre and post menopausal women (13) and a 30% difference in PON1 activity between AA and CC genotypes (16) we would need 9 subjects per genetic group. Therefore, considering the distribution of the three genotypes (28% for the minor frequency TT genotype (target n = 10), 41% for CT (target n = 14) and 31% for the CC genotype (target n = 11) (16) we would need a total of 35 women per menopausal stage considering the genotype distribution. After applying the inclusion and exclusion criteria described, 76 women were enrolled in the study, and divided into two groups: premenopausal (n = 40) and postmenopausal (n = 36). Women with no menstrual activity in the last twelve months and older than 40 years were considered as postmenopausal (23).

### Data and blood collection

A general questionnaire was applied for the evaluation of health conditions, level of education, use of medications and life habits. Additionally, food intake was assessed by application of a food frequency questionnaire, containing 83 foods components, following the standards previously described (24). Food were classified as processed (40 types of food), *in natura* (26 types of food) and rich in cholesterol/saturated fatty acids (32 types of food), as proposed by the New Food Guide for the Brazilian Population (25). For each food, a weekly consumption frequency was established and from this score the consumption medians were established for each of the 3 categories (processed, *in natura*, and rich in cholesterol/fatty acids) and patients classified as ingesting above or below the average. In addition, based on the composition of food and ingestion amount reported daily average intake of calorie, protein, carbohydrate, lipid and fiber were calculated.

Body mass (kg) and height (centimeters) were measured on a mechanical scale (Welmy, Santa Bárbara d’Oeste, SP, Brazil). The body mass index (BMI) of each participant was obtained through the equation body mass (kg) divided by stature squared (m) and interpreted according to the classification of the World Health Organization (26).

Blood collection was scheduled at the time of the interview within a two-week interval. About 5 mL of blood from each patient was collected by venipuncture, after a 12-hour fasting period, in dry vials for biochemical measurements. About 1 mL of blood was collected in an EDTA coated vial for DNA extraction.

### Biochemical analysis

PON1 activity was evaluated by the measurement of the arylesterase activity according to previously described (16,27). The activity was measured from the rate of phenol formation by monitoring the absorbance increase at 270 nm. The working reagent consisted of 20 mM Tris/HCl, pH 8.0, containing 1 mM CaCl_2_ and 4 mM phenylacetate as substrate. Samples were diluted 1:3 in buffer before added to the working reagent and the change in absorbance was recorded for 60 sec. One unit of arylesterase activity was considered equal to 1 mM of phenol formed per minute. The activity is expressed in U/mL. Blank samples containing water were used to correct non-enzymatic hydrolysis.

Blood samples were also sent to a clinical laboratory for analysis in an automated biochemical analyzer. Total cholesterol (TC), HDL cholesterol (HDL), LDL cholesterol (LDL), triglycerides, and glucose were measured by automated enzymatic methods. Glycated hemoglobin (HbA1c) was determined by the immunoturbidimetry method.

### Genotyping

To extract the genomic DNA, blood samples were processed following an adapted protocol (28). For the determination of the *PON1* T(-107)C genotype the procedures were followed as previously described (15,16). Briefly, the amplification of the region where the SNP is located was performed by PCR, using 10 μL of GOTaq^®^ mix for PCR (Promega, Madison, WI, USA), 1 μL (10 μM stock concentration) of the forward primer AGCTAGCTGCGGACCCGGCGGGGAGGaG and 1 μL of the reverse primer GGCTGCAGCCCTCACCACAACCC. Standard conditions for the PCR reaction were used, with an annealing temperature of 67 °C. The lower-case letter in the forward primer indicates a mismatch that introduces a restriction site for the *BsrB*I enzyme (New England BioLabs, Cambridge, UK), as there is no specific restriction site cleaving the original DNA sequence. After digestion of the PCR product for 2 h at 37 °C, the DNA fragments were separated by 2% agarose gel electrophoresis, stained with SYBR Safe (Applied Biosystems, Foster City, CA, USA). The C allele was identified by fragments of 28 and 212bp, while the T allele resulted in the undigested 240bp fragment. We were unable to process seven samples for DNA genotyping.

### Data processing and analysis

Statistical analyzes were performed on SAS University Edition (SAS, Cary, NC, USA) and figures were generated in Graphpad Prism 5 (GraphPad, La Jolla, CA, USA). All parameters had passed the preliminary Shapiro-Wilk test for normality. Comparisons between the pre and postmenopausal women were made using the MIXED MODEL procedure using ethnicity and BMI as co-variates. For testing individual effects of menopause, genotype and food consumption on PON1 activity as well as its interactions, the same MIXED MODEL procedure was used. Post-hoc analysis was performed using the Tukey adjustment. Additionally, to verify the linear or quadratic effect of the presence of the C allele on PON1 activity, the GLM model was used, considering the genotypes TT as 0, CT as 1 and CC as 2. The GLM linear effect was also used to test the effect of the level of consumption of processed, *in natura* and cholesterol/fatty acids rich diets on serum PON1 activity. In the unadjusted analysis, simple linear regression was used to compare PON1 activity according to genotype. Multivariable analyses included linear regression using three different models. Model one included BMI and total dietary energy content; Model 2 included only the total lipid and cholesterol content of the diet and Model 3 included BMI, total dietary energy content, total dietary lipid and cholesterol content. In order to analyze the effect of several variables on the association between serum PON1 activity and genotype, we ran models including one extra variable at a time, and examined the change in beta (β) coefficient and coefficient of determination (R^2^). The order in which variables were included in the model was defined by the significance of its association with serum PON1 activity. Hardy-Weinberg equilibrium and comparison of genotype distribution between pre and postmenopausal women were compared by the Chi-square test. The level of significance was set at p ≤ 0.05.

## RESULTS

The general characteristics of the study population are presented in Table 1. Postmenopausal women were older (p < 0.0001), as expected, but there was no difference in BMI distribution among groups. Additionally, a higher proportion of postmenopausal women were currently smokers (p = 0.04).

**Table 1:** General characteristics of women in the pre and postmenopausal stages recruited for the study.

Parameter	Premenopausal	Postmenopausal	p value
Sample size	40	36	-
Age (years)^1^	42.0 ± 0.8	53.5 ± 0.6	< 0.0001
Age at menopause	-	46.5 ± 0.7	-
Use of contraceptives	34.2% (13/38)	-	
Ethnicity^2^			
White	70.0% (28/40)	86.1% (31/36)	0.11
Mixed/Black	25.0% (10/40)	11.1% (4/36)	0.15
Undeclared	5.0% (2/40)	2.8% (1/36)	-
Smoking^2^			
Currently	5.4% (2/37)	22.8% (8/35)	0.04
Past	18.9% (7/37)	8.6% (3/35)	0.30
BMI^3^			
Normal range (18-25)	17.5% (7/40)	27.8% (10/36)	0.51
Overweight (25-30)	15.0% (6/40)	16.7% (6/36)	
Obese (> 30)	67.5% (27/40)	55.5% (20/36)	

^1^ Means are presented as least square means ± standard error of the mean and where compared using the MIXED model, using BMI and ethnicity as co-variates and the Tukey post-hoc test, ^2^ Proportions are presented as percentages and where compared using Chi-square test, ^3^ Body mass index.

Regarding the metabolic profile, glucose (p = 0.03), HbA1c (p = 0.002) and total cholesterol (p = 0.002) concentrations were higher in post than premenopausal women (Table 2). There were no differences for BMI, HDL and LDL cholesterol, triglycerides and PON1 activity between pre and postmenopausal women (p > 0.05; Table 2). Additionally, the use of contraceptives did not affect serum PON1 activity (Yes: 73.8 ± 4.7 U/mL, No: 82.5 ± 7.3 U/mL, p = 0.32).

**Table 2:** Blood biochemistry parameters for women in the pre and postmenopausal stages

Parameter	Premenopausal^1^	Postmenopausal^1^	p value
Glucose (mg/dL)	84.6 ± 4.3	99.2 ± 4.5	0.03
HbA1c (%)^2^	5.4 ± 0.2	6.2 ± 0.2	0.002
Total Cholesterol (mg/dL)	182.6 ± 6.2	211.6 ± 6.7	0.002
HDL (mg/dL)^3^	48.8 ± 2.2	53.4 ± 2.3	0.16
LDL (mg/dL)^4^	107.6 ± 6.0	128.7 ± 7.6	0.07
Triglycerides (mg/dL)	138.3 ± 13.1	152.6 ± 14.2	0.46
PON1 (U/mL)^5^	74.4 ± 4.4	81.3 ± 4.7	0.28

^1^ Means are presented as least square means ± standard error of the mean and where compared using the MIXED model, using BMI and ethnicity as co-variates and the Tukey post-hoc test, ^2^ Glycated hemoglobin, ^3^ High-density lipoprotein cholesterol, ^4^ Low-density lipoprotein cholesterol, ^5^ Paraoxonase 1.

**Figure 1:**
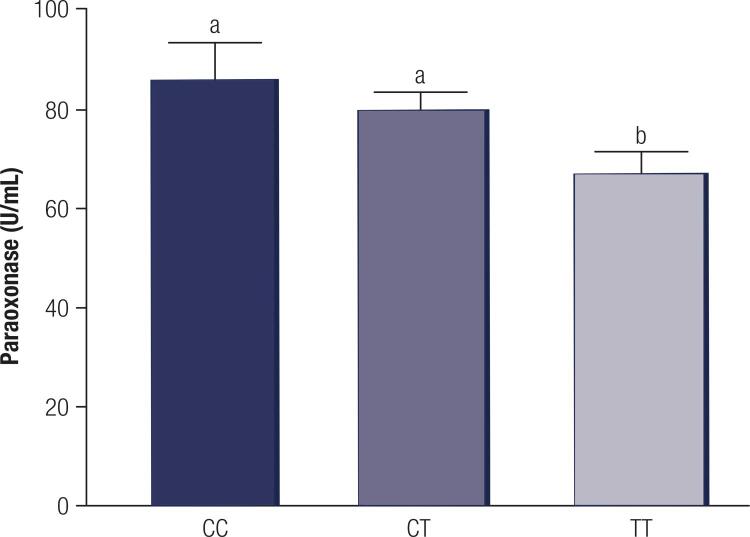
Paraoxonase 1 (PON1) activity (U/mL) among women from the CC, CT and TT genotypes for the *PON1* T(-107)C polymorphism. The data are presented as the means ± SEM. Different letters indicate significant differences at p < 0.05.

The genotype frequency for the *PON1* C(-107)T SNP is presented in Table 3. The allelic frequency for the C allele was 46.4% and for the T allele it was 53.6%. The population was in Hardy-Weinbeg equilibrium (p = 0.29), and genotype distribution is presented in Table 3. There was no overall difference in genotype distribution between pre and post-menopausal women in the chi-square test (p = 0.22). Regarding the individual effects of genotype and menopause on PON1 activity, there was an effect of the genotype (Figure 1; p = 0.03), but no effect of menopause (p = 0.20) and also no significant menopause by genotype interaction (p = 0.83). There was a linear effect of the presence of the C allele (p = 0.003), where carriers of the C allele had higher PON1 activity (CC: 88.9 ± 6.5 U/mL and CT:79.9 ± 4.7 U/mL) than women of the TT genotype (66.6 ± 5.9 U/mL) (p < 0.05). There was no effect of the genotype on concentrations of glucose, HbA1c, total cholesterol, HDL, LDL and triglycerides (p > 0.05).

**Table 3:** Genotype frequency distribution for the *PON1* T(-107)C in women in the pre and postmenopausal stages

Genotype	Overall	Premenopausal	Postmenopausal	p value
CC	24.6% (17/69)	21.6% (8/37)	28.1% (9/32)	1.00
CT	43.5% (30/69)	37.8% (14/37)	50.0% (16/32)	0.33
TT	31.9% (22/69)	40.4% (15/37)	21.9% (7/32)	0.12

Proportions are presented as percentages and where compared using Chi-square test.

Consumption of calories, macronutrients, cholesterol and fiber for each group of pre and postmenopausal women is shown in Table 4. Premenopausal women had a higher intake of calories, protein and carbohydrate than postmenopausal women (p < 0.05), but no differences were observed for fat and cholesterol intake (p > 0.05). Additionally, when categorizing foods in processed, *in natura* and rich in cholesterol/SFA, we observed no difference in the frequency of consumption of these types of food between pre and postmenopausal women (Table 4; p > 0.05). There was a weak association of the levels of *in natura* food consumption with serum PON1 activity (p = 0.04, R^2^ = 0.06); however, there was no effect of consumption of processed (p = 0.25) or cholesterol/SFA foods (p = 0.11) on PON1 activity.

**Table 4:** Daily estimated profile of food intake in patients in the pre and postmenopausal stages

Parameter	Premenopausal	Postmenopausal	p value
Calories (kcal)	1849.5 ± 104.5	1541.2 ± 115.8	0.02
Carbohydrates (g)	223.1 ± 17.4	165.7 ± 20.1	0.01
Protein (g)	109.3 ± 8.5	84.9 ± 9.8	0.03
Lipids (g)	58.4 ± 2.7	52.8 ± 3.0	0.12
Cholesterol (mg)	108.8 ± 11.3	95.3 ± 13.1	0.35
Processed^1^	1.8 ± 0.1	1.6 ± 0.1	0.16
*In natura*^1^	2.2 ± 0.2	2.4 ± 0.2	0.45
Fat/Cholesterol^1^	1.2 ± 0.1	1.0 ± 0.1	0.28

Means are presented as least square means ± standard error of the mean and where compared using the MIXED model, using BMI and ethnicity as co-variates and the Tukey post-hoc test.

^1^ Weekly ingestion frequency of each class of food.

The crude and adjusted analyzes of the association between PON1 activity and genotype interaction with intake profile are presented in Table 5. The enzyme variation was estimated at 0.12 (12.0%) by adjusted determination coefficients (R^2^) with inclusion of total diet lipid content and dietary cholesterol in the multiple linear regression model (model 2) (p < 0.05). In Table 6 the analysis of the mediators of PON1 change according to menopausal stage is demonstrated. BMI and total dietary lipid content when associated to PON1 genotype were the main factors for enzyme variation in postmenopausal women, with 0.12 (12.0%) and 0.31 (31.0%) of R^2^, respectively (p < 0.05). An equivalent analysis was performed for premenopausal women and a similar pattern regarding lipid content was observed (p > 0.05).

**Table 5:** Unadjusted and adjusted analyzes of the association between PON1 activity and genotype interaction with body mass index and food intake profile

Genotype	B	SE	β	R^2^	P-value	PON1-EV
	**Unadjusted**					
TT	66.95	5.31	REF	0.08	REF	REF
CC	18.61	8.04	0.32		0.020	85.56
CT	12.42	7.04	0.24		0.083	79.37
	**Model 1^a^**					
TT	60.96	15.23	REF	0.10	REF	REF
CC	17.62	8.32	0.30		0.038	78.58
CT	11.59	7.04	0.22		0.123	72.55
	**Model 2^b^**					
TT	58.78	9.45	REF	0.12	REF	REF
CC	17.36	8.08	0.30		0.036	76.14
CT	13.23	7.11	0.26		0.068	72.01
	**Model 3^c^**					
TT	63.70	14.99	REF	0.17	REF	REF
CC	13.78	8.30	0.24		0.105	77.48
CT	11.61	7.26	0.23		1.115	75.31

B: regression coefficient; SE: standard error; R^2^: adjusted determination coefficient; β: beta coefficient; PON1 EV: estimated value of Paraoxonase 1.

^a^ Adjusted for BMI and total dietary energy content, R^2^ p = 0.147, ANOVA;

^b^ Adjusted for total dietary lipid and cholesterol content, R^2^ p = 0.097, ANOVA;

^c^ Adjusted for BMI, total dietary energy, lipid and cholesterol content, R^2^ p = 0.079, ANOVA.

**Table 6:** Adjusted analyzes of the association between PON1 activity and genotype interaction with food intake profile for pre and postmenopausal women

Parameter (Model)	B	SE	β	R^2^	p-Value
All participants (n = 66)					
Genotype (1)	67.50	3.90	0.31	0.09	0.010
1+BMI (2)	0.37	0.35	0.13	0.11	0.028
2+Energy (3)	-0.03	0.00	-0.12	0.12	0.046
3+Total fat (4)	0.77	0.45	0.45	0.16	0.029
4+Cholesterol (5)	0.07	0.06	0.15	0.18	0.034
Premenopausal (n = 34)					
Genotype (1)	67.86	5.15	0.25	0.06	0.136
1+BMI (2)	-0.18	0.50	-0.06	0.06	0.362
2+Energy (3)	0.00	0.00	-0.12	0.08	0.486
3+Total fat (4)	0.49	0.50	0.35	0.11	0.499
4+Cholesterol (5)	0.18	0.08	0.47	0.24	0.148
Postmenopausal (n = 32)					
Genotype (1)	66.86	13.47	0.34	0.12	0.050
1+BMI (2)	1.10	0.52	0.34	0.24	0.019
2+Energy (3)	0.00	0.01	-0.07	0.24	0.047
3+Total fat (4)	2.00	1.22	0.88	0.31	0.033
4+Cholesterol (5)	0.05	0.10	0.08	0.32	0.063

B: regression coefficient; SE: standard error; R^2^: adjusted determination coefficient; β: beta coefficient.

## DISCUSSION

In this study we investigated changes in serum PON1 activity in pre and postmenopausal women and its association with the *PON1* C(-107)T SNP and food consumption during those stages. It was observed that the TT genotype carriers had lower serum PON1 activity than the CT and CC genotypes carriers. However, menopause did not affect serum PON1 activity and there was no combined effect of the genotype and the menopause status on serum PON1 activity. Despite that, postmenopausal women had higher levels of glucose and total cholesterol. Total fat and cholesterol contributed with the genotype to affect serum PON1 activity.

We did not found difference in PON1 activity between pre and postmenopausal women which is in agreement with a previous report (29). Despite that, some have reported decreased serum PON1 activity after surgically induced menopause in women, which was restored by the estrogen replacement therapy (13), suggesting estradiol as an important PON1 regulator. In fact, it is suggested that women using oral contraceptives have increased PON1 activity (30), but we did not observe any effect of the contraceptive use on serum PON1 activity in the current study. This lack of difference can be related to the age of the participants, which were all older than 35 years, and already have declining estradiol concentrations (31). Even though PON1 activity did not change between pre and postmenopausal women, it was observed an increase in total cholesterol and also a tendency for higher LDL concentrations. These changes in the lipid profile were previously demonstrated by others (29). The high prevalence of obesity associated with higher cholesterol and glucose concentrations in these postmenopausal women are important risk-factors for chronic diseases such as hypertension and type II diabetes, which also contribute to the occurrence of CVD.

The majority of women in the present study was from the *PON1*(-107) CT genotype, and a smaller proportion was from the CC genotype, which was associated with the highest levels of PON1 activity. This distribution is similar to previous findings (15), including in a study with a group of women from southern Brazil (16). Overall, women carrying the C allele had higher PON1 activity than women of the TT genotype, as observed before (15,16). Despite this, PON1 activity was not different between menopause and pre menopause stage and this lack of difference was independent of the genotype. Since menopause is associated with several biochemical and endocrine changes (3,4), we could expect that it would challenge PON1 activity differently for women carrying different genotypes. However, in the current study this was not observed and the results suggest that PON1 activity remains unchanged after menopause, being mainly regulated by the PON1 T(-107)C genotype.

Others had shown interactions of the diet and lifestyle with the *PON1* genotype (16,32). In this study, no major influence of lipid and processed foods on PON1 activity were observed when categorizing women by median food intake. However, the model predicting PON1 activity was slightly better when genotype, total fat and cholesterol content in the diet were all included. This was more evident when considering only post-menopausal women. These findings are in agreement with previous observations in larger populations, indicating that the main modulator of PON1 activity is the genotype and not the diet or lifestyle (21). Others (21) working with very large group of subjects found a contribution lower than 10% of the diet over serum PON1 activity, with the majority of the effects remaining unexplained, and also found genetics as the main regulator. Among the diet effects, some found that the type of lipids and fatty acids have a significant effect on PON1 activity (33), as we currently observed. Previous studies had also found an interaction between food consumption and the *PON1* genotype in women (16,32). This is further indication of the complexity of the modulation of serum PON1 activity, with many factors regulating its activity and interacting with the genotype. Although the menopausal stage had no effect in PON1 activity, it seems that this group was slightly more susceptible to the effects of the diet. It is well known that high consumption of fruits and vegetables is associated with lower CVD risk in women (34), and as PON1 is critical for prevention of CVD it is expected that it will also be increased by this type of food. Indeed, in type 2 diabetes patients, higher fruits and vegetables intake increased the activity of enzymes associated with the antioxidant properties of HDL, including PON1 (35), which is in alignment with our findings over consumption of *in natura* foods.

In sum, we observed the *PON1* C(-107)T genotype as the major regulator of serum PON1 activity. We did not observe any effect of the menopausal stage on serum PON1 activity. However, the model predicting PON1 activity was slightly better when genotype, total fat and cholesterol content in the diet were all considered together, especially for postmenopausal women. The lipid and glycemic profile were altered in postmenopausal women, which associated with increased BMI, can predispose this population to the development of CVD and diabetes. Our findings should be considered carefully as women in this study had different intervals between menopause and the moment of sample collection, which can shift the trends observed.
